# Transcriptome Analysis of Triple Mutant for OsMADS62, OsMADS63, and OsMADS68 Reveals the Downstream Regulatory Mechanism for Pollen Germination in Rice (*Oryza sativa*)

**DOI:** 10.3390/ijms23010239

**Published:** 2021-12-27

**Authors:** Eui-Jung Kim, Woo-Jong Hong, Yu-Jin Kim, Ki-Hong Jung

**Affiliations:** 1 Graduate School of Biotechnology & Crop Biotech Institute, Kyung Hee University, Yongin-si 17104, Korea; alice804@khu.ac.kr (E.-J.K.); hwj0602@khu.ac.kr (W.-J.H.); 2 Department of Life Science and Environmental Biochemistry, and Life and Industry Convergence Research Institute, Pusan National University, Miryang-si 50463, Korea

**Keywords:** *Oryza sativa*, MADS, pollen germination, starch, cell wall, CRISPR-cas

## Abstract

The MADS (MCM1-AGAMOUS-DEFFICIENS-SRF) gene family has a preserved domain called MADS-box that regulates downstream gene expression as a transcriptional factor. Reports have revealed three *MADS* genes in rice, *OsMADS62*, *OsMADS63*, and *OsMADS68*, which exhibits preferential expression in mature rice pollen grains. To better understand the transcriptional regulation of pollen germination and tube growth in rice, we generated the loss-of-function homozygous mutant of these three *OsMADS* genes using the CRISPR-Cas9 (clustered regularly interspaced short palindromic repeats-CRISPR associated protein 9) system in wild-type backgrounds. Results showed that the triple knockout (KO) mutant showed a complete sterile phenotype without pollen germination. Next, to determine downstream candidate genes that are transcriptionally regulated by the three *OsMADS* genes during pollen development, we proceeded with RNA-seq analysis by sampling the mature anther of the mutant and wild-type. Two hundred and seventy-four upregulated and 658 downregulated genes with preferential expressions in the anthers were selected. Furthermore, downregulated genes possessed cell wall modification, clathrin coat assembly, and cellular cell wall organization features. We also selected downregulated genes predicted to be directly regulated by three *OsMADS* genes through the analyses for promoter sequences. Thus, this study provides a molecular background for understanding pollen germination and tube growth mediated by *OsMADS62*, *OsMADS63*, and *OsMADS68* with mature pollen preferred expression.

## 1. Introduction

Plants use pollen grains (the male gamete) to deliver sperm cells to the ovary. When the pollen maturing inside the anther reaches the tricellular stage, anthesis and dehiscence occur, and pollen grains are released [1]. To prevent protein damage in this process, the pollen walls fold to maintain a dehydrated state and become temporarily dormant. In addition, when the dehydrated pollen grain blows in the wind and settles in the stigma, it absorbs surrounding water to regain metabolic activity and prepares for pollen germination [2,3]. In the case of rice (*Oryza sativa*), the viability of pollen disappears if it fails to stick to stigma within five minutes, and since it takes less than ten minutes from germination to tube burst in an in vitro environment, quick and sophisticated mechanisms are required for successful fertilization [4,5,6]. Furthermore, pollen grains should have enough proteins and energy for successful germination and tube growth. However, the synthesis and accumulation of starch are primarily required to achieve this [7]. Mature pollen contains starch as polysaccharides, sucrose as disaccharides, and glucose as monosaccharides, mainly storing energy sources in the form of starch [7]. Sucrose and starch are present inside the dehydrated pollen grain right after anthesis, and energy generated by starch decomposition is used for pollen germination [8]. An appropriate amount of starch must be accumulated in the final stage of pollen maturation, and problems in this process lead to the male-sterile phenotype. The loss-of-function of *OsHXK5* encoding hexokinase significantly reduces starch content in pollen and shows male sterility [9]. Furthermore, the mutation of *OsSUT1* encoding a sucrose transporter shows a defect in pollen germination and cannot produce any seeds [10,11]. In addition, the mature pollen grain must have proteins related to cell wall synthesis and vesicular transport processes, such as exocytosis and endocytosis. The pollen wall is divided into two layers; exine and intine. The exine is composed of sporopollenin, intine is made of cellulose and pectin, but the pollen tube wall is made of a single layer of pectin [12]. Pectin methylesterases (PME) induce pollen wall hardness by demethylesterification of homogalacturonan (HG) pectin component; pectin methylesterases inhibitor (PMEI) inhibit PME and methylesterifies pectin, thereby relaxing the pollen walls to induce elongation [13,14,15]. Thus, for pollen tube bulging, the cell wall should be relaxed by the interaction between PME and PMEI. Additionally, for rice, 11 *OsPMEs* and 13 *OsPMEI* have specifically high expression in the pollen tissue, and exogenic PME treatment induces pollen tube hardness to prevent elongation [16].

MADS-box protein is a transcription factor (TF) with a highly conserved MADS domain and essential functions in floral organ development, seed development, floral organ identity determination, and regulation of flowering time [17,18,19,20]. It regulates the expression of downstream genes by directly binding to the CArG motif of the promoter. MADS domain protein is mainly divided into SRF-like MADS domain (Type 1) and MEF2-like MADS domain protein (Type 2). Among the MEF2-like types (also known as MIKC-type), the MIKC*-type with a longer intervening domain (I) and duplication of keratin-like domains (K) is well known for its function in pollen development [21]. The MADS-box protein could be dimerized to activate the function as a TF [22]. In the case of *Pteridophytes*, such as *Selaginella moellendorffii* and *Shorea pallescens*, the two groups in S- and P-clades, belonging to MIKC*, mainly form dimers and function as TF [23]. In *Arabidopsis thaliana*, there have also been studies on *AGAMOUS-LIKE (AGL)* genes which belong to one of the types of MADS-box. There are five *AGL* genes (*AGL30, AGL65, AGL66, AGL94, and AGL104*), which are preferentially expressed in the pollen [24], especially at the tricellular stage after the end of the second mitotic division [25,26]. Among them, three combinations of AGL30/66, AGL65/66, and AGL65/104 form dimers and function as TF [26]. MADS directly regulates transcriptional levels by binding to the CArG motif (CC[A/T]6GG) in the promoters of the downstream genes [27,28]. Although MADS binds to the serum response element (SRE)-type CArG box, CC[A/T]6GG, it can be attached to MEF2-CArG box, C[A/T]8G or CTA[A/T]4. For example, in *Arabidopsis thaliana*, AGL30/66, AGL65/66, and AGL65/104 dimerization forms have a higher preference for the MEF2-CArG box. In addition, genes specifically expressed in pollen at the tricellular stage are better preserved in MEF2-CArG boxes than in the SRE-CArG boxes based on their 3 kb upstream promoter [29].

In the case of *Oryza sativa*, *OsMADS62*, *OsMADS63*, and *OsMADS68* have specifically high expression in the pollen grains at the tricellular stage; *OsMADS62* and *OsMADS63* belong to the MIKC* S-clade, whereas *OsMADS68* belongs to the MIKC* P-clade [30]. These three proteins can form homodimers and heterodimers through diverse combinations, and the interactions of *OsMADS62/68* and *OsMADS63/68* are the strongest [30]. Pollen viability and germination defects were identified in RNAi mutants with reduced *OsMADS62* or *OsMADS68* expression in the *osmads63* mutant background [30]. However, the defects were observed at the heterozygote state with a mixture of normal and defective pollens. Thus, detailed phenotypic observation and related regulatory mechanisms have not been sufficiently investigated. Here, we generated homozygous triple loss-of-function mutants of three *OsMADS (box)* genes belonging to the MIKC*-type (*OsMADS62*, *OsMADS63*, and *OsMADS68*) that are preferentially expressed in mature pollen grains. By analyzing the total RNA of mature anther through RNA-seq analysis, we identified differentially expressed genes (DEG) in the triple loss-of-function mutant anther versus wild-type anther. Furthermore, we conducted functional classification analyses using gene ontology (GO) enrichment, MapMan, and Kyoto Encyclopedia of Genes and Genomes tools. Down(-regulated) DEGs are closely associated with biological processes for pollen tube development, such as starch accumulation and cell wall synthesis. Because *OsMADS62*, *OsMADS63*, and *OsMADS68* function in the late stage of pollen maturation, germination, and tube elongation, transcriptome analysis using triple mutations should provide an important molecular basis for future research.

## 2. Results

### 2.1. OsMADStri Pollen Cannot Germinate, Resulting in a Male-Sterile Phenotype

**Figure 1:**
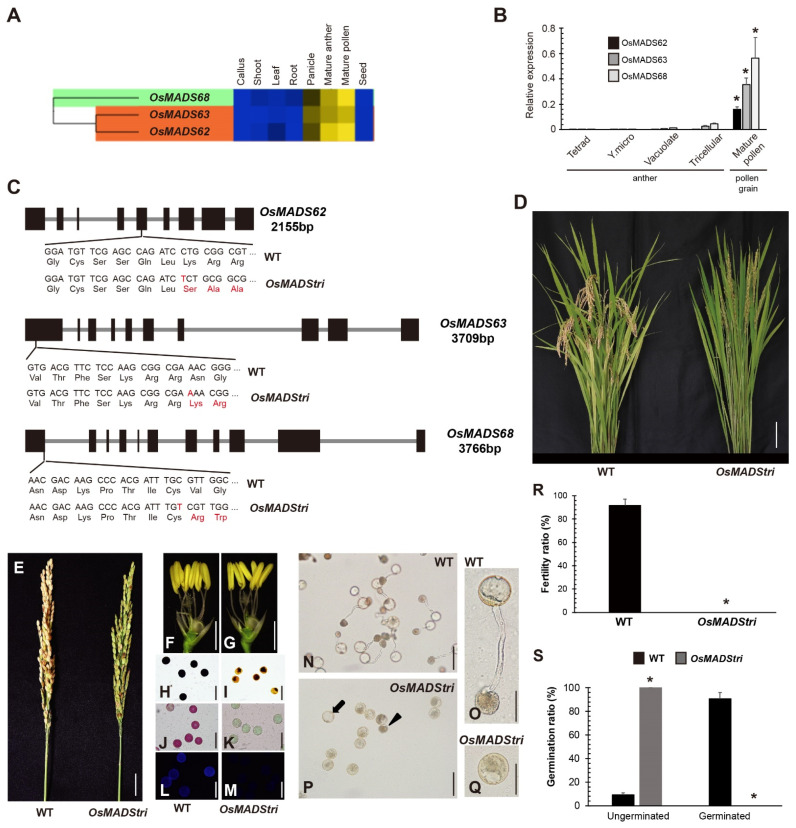
The mutation target site and phenotype of *OsMADStri*. (**A**) Gene expression pattern of three *OsMADS* using CAFRI-rice. Leaf, 7 days before flowering to 7 days after flowering; Shoot and Root, 7 days after germination; Panicle, 7 days before flowering; Mature anther, anther at anthesis period. Heatmap displays the expression value according to the color gradation. Blue is the lowest, and yellow is the highest expression value. (**B**) The transcriptome expression pattern of the three *OsMADS* genes in anther at various developmental stages and pollen grains after anther dehiscence. Tetrad, the anther that contains mitotic pollens at late tetrad stage; Y. micro, the anther that contains pollens at young microspore stage; Vacuolate, the anther that contains pollens at vacuolated microspore stages; Tricellular, mature anther with trinucleate pollen; Mature pollen, the pollen grains right after anther dehiscence. Error bars indicate the standard deviation and *p*-value calculated by one-way Analysis of Variance (ANOVA). *, *p* < 0.01. (**C**) *OsMADS62*, *OsMADS63*, and *OsMADS68*, the target genes of *OsMADStri*. The gene size is written below its name by base pairs. The fifth exon of *OsMADS62* was a target site, and thymine was inserted. The first exon of *OsMADS63* and *OsMADS68* were mutated by one base insertion, respectively. bp, base-pair; WT, wild-type. (**D**) The phenotype of the whole plant. Bars = 10 cm. (**E**) The panicle pictures of WT and *OsMADStri*. Bars = 2 cm. (**F**,**G**) The pictures of reproductive tissue of WT and *OsMADStri*. Bars = 2 mm. (**H**–**M**) Histochemical staining of the mature pollen grain of WT and *OsMADStri*. (**H**,**I**) is the iodine potassium iodide staining, (**J**,**K**) is Ruthenium red staining, (**L**,**M**) is Calcofluor white staining. Bars = 80 µm. (**N**–**Q**) In vitro germination is performed in solid pollen germination media. Black arrow and triangle indicate hydrated and unhydrated pollen grains, respectively. Bars = 80 µm, 40 µm. (**R**,**S**) Fertility ratio and germination ratio of WT and *OsMADStri*. Error bars indicate the standard deviation and *p*-value calculated by one-way Analysis of Variance (ANOVA). *, *p* < 0.01.

The recent transcriptome analysis identified three *MADS-box* genes in rice, *OsMADS62*, *OsMADS63*, and *OsMADS68*, which had a high expression in mature anther and pollen grains [30]. In the CAFRI-rice database, the three abovementioned genes showed similar expression patterns: weak expression in panicle, intermediate expression in mature anther, and strong expression in mature pollen grains (Figure 1A). However, these three *OsMADS* genes showed low expression in the anthers at the late tetrad, young microspore, and vacuolated microspore stages (Figure 1B). However, the expression increased a little in mature anther, including tricellular pollen, and was the strongest in pollen grain, right after anther dehiscence. Among three *OsMADS* genes, *OsMADS68* had the highest expression and *OsMADS62* had the lowest expression. Furthermore, *OsMADS62* and *OsMADS63* shared higher sequence similarities compared with *OsMADS68*. It has been reported that the three genes have functional redundancy on late pollen development. For overcoming the functional redundancy and the functional analysis of these genes, we generated the loss-of-function homozygous triple mutants by targeting three genes simultaneously using the CRISPR-Cas9 (clustered regularly interspaced short palindromic repeats-CRISPR associated protein 9) system. The first exons of *OsMADS63* and *OsMADS68* and the fifth exon of *OsMADS62* were targeted for gene editing. One base (thymine) was inserted in the fifth exon of *OsMADS62* and the first exon of *OsMADS68*, and one base (adenine) was also inserted in the first exon of *OsMADS63*; this loss-of-function mutation was named *OsMADStri* (Figure 1C). All mutations resulted in an early stop codon, causing an abnormal protein. The T0 generation of the *OsMADStri* had a sterile phenotype without seeds. Unlike the wild-type (WT) Dongjin plants that produce seeds over 90%, the T0 generation of *OsMADStri* cannot produce seeds and showed a complete sterile phenotype (Figure 1D,E,R). The reciprocal crossing assay with control WT plants showed that *OsMADStri* has a defect in developing male reproductive tissues. When *OsMADStri* is used as the pollen donor, the control plants cannot produce any seeds, but it can produce heterozygous progeny when used as the pollen recipient (Figure S1). *OsMADStri* did not show any difference in anther size compared with the WT (Figure 1F,G). However, in the case of iodine potassium iodide solution ($I_{2}KI$) staining, the OsMADStri pollen grains were not stained at the tricellular stage (Figure 1H,I). Likewise, the difference could be observed in Ruthenium red and Calcofluor white staining (Figure 1J–M). This indicates that acidic polysaccharides constituting pectin and cellulose of the intine layers of *OsMADStri* were abnormal. The size and shape of *OsMADStri* mature pollen grains seemed identical to those of the WT. However, the *OsMADStri* pollen showed abnormal germination patterns and could not elongate any pollen tube under in vitro germination (Figure 1N–Q,S). As the defects were identified at the late pollen development for male–female organ interaction, we expect these three MADS-box TFs to play key regulatory roles in the developmental process.

### 2.2. Transcriptome Analysis Suggests the Potential Downstream Genes of OsMADS62, OsMADS63, and OsMADS68

**Figure 2:**
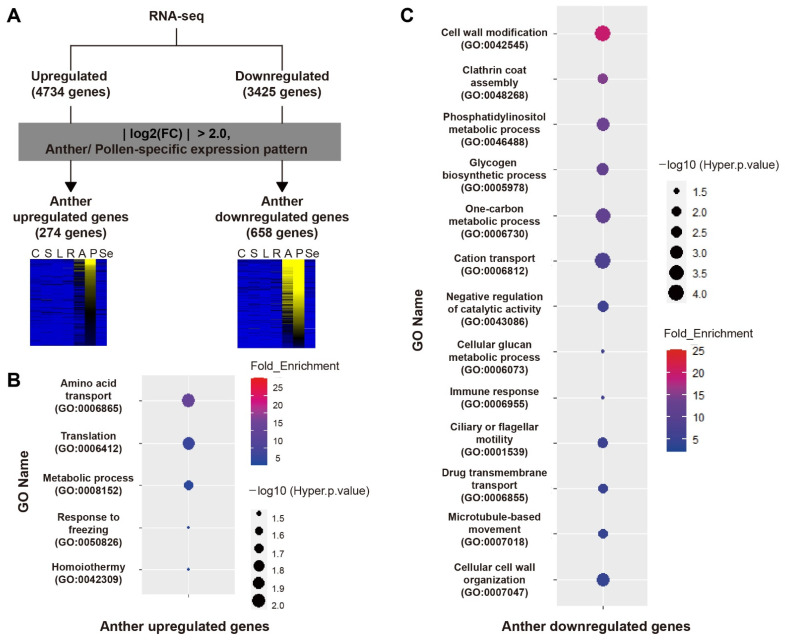
Schematic of DEG screening and GO annotation graph. (**A**) This schematic diagram shows how we screened Differentially Expressed Genes in mature pollen grains (pollen DEGs) after conducting RNA-seq. Four thousand, seven hundred and thirty-four upregulated genes and 3425 downregulated genes were selected, and the expression patterns of each tissue were compared, and the absolute value of $log_{2}\left(FC\right)$ was 2.0 or higher was selected in detail. Thus, 274 genes were finally selected as pollen upregulated genes and 658 genes as pollen downregulated genes. The heatmap was drawn based on the RNA-seq value of each tissue in wild-type plants, yellow indicates a high expression, and blue indicates a low expression. C, Callus; S, Shoot; L, Leaf; R, Root; A, Anther; P, Pollen grains, Se, Seed. (**B**,**C**) GO enrichment annotation of pollen DEGs. The color represents the fold enrichment of GO, and the size of the circle represents the hyper *p*-value. The GO number was written under GO Names (terms), sorted in descending order based on the fold enrichment.

We tried to identify downstream genes of *OsMADS62*, *OsMADS63*, and *OsMADS68* using WT and *OsMADStri* line anthers. RNA-seq analysis was performed with the anthers sampled at the tricellular stage of WT and *OsMADStri*. Differentially expressed genes (DEG) were selected based on three criteria; (1) genes with absolute log_2_ value (*OsMADStri* anther/WT anther; fold change/FC) was ≥1, (2) genes which *p*-value of log_2_ (FC) was ≤0.05 in three biological replicates, and (3) the mean expression value in WT tricellular anther sample was ≥10. Out of the total 55,986 rice genes, there were 8159 DEGs: 4734 upregulated genes and 3425 downregulated genes (Figure 2A). Additionally, DEGs were refined by selecting (1) genes having preferential expression in mature anthers and pollen grains compared with eight other organs, callus, leaf, root, seed, shoot, pre-flowering panicle, anther, and pollen grains, through K-means clustering analysis and (2) genes for which absolute value of $log_{2}\left(FC\right)$ was ≥2. DEGs were selected based on their anatomical expression patterns because the three *OsMADS* genes had a highly specific expression in mature anther and pollen grains. The DEGs selected were expressed not only in wild-type anther but also in wild-type pollen grain. As a result, 274 genes were selected as anther (preferred) upregulated genes, and 658 genes were selected as anther (preferred) downregulated genes (Figure 2A).

### 2.3. Downregulated Genes in OsMADStri Anther/Pollen Mainly Show GO Enrichment Related to Cell Wall Biosynthesis and Vesicle Secretion Processes

GO enrichment analysis was conducted to identify biological processes closely associated with these mature anther DEGs, and results are as follows: amino acid transport (GO:0006865), translation (GO:0006412), and metabolic process (GO:0008152) were the most enriched in anther upregulated genes. However, cell wall modification (GO:0042545), clathrin coat assembly (GO:0048268), and phosphatidylinositol metabolic process (GO:0046488) were the most enriched in anther downregulated genes (Figure 2B,C). Interestingly, GOs, such as clathrin coat assembly (GO:0048268), cation transport (GO:0006730), and microtubule-based movement (GO:0007018), were only enriched in anther downregulated genes (Figure 2C). They are mainly related to endocytosis/exocytosis, which is essential for pollen tube elongation.

### 2.4. MapMan Analysis for Downregulated Genes in OsMADStri Further Supports the Significance of Genes Related to Cell Wall Synthesis

**Figure 3:**
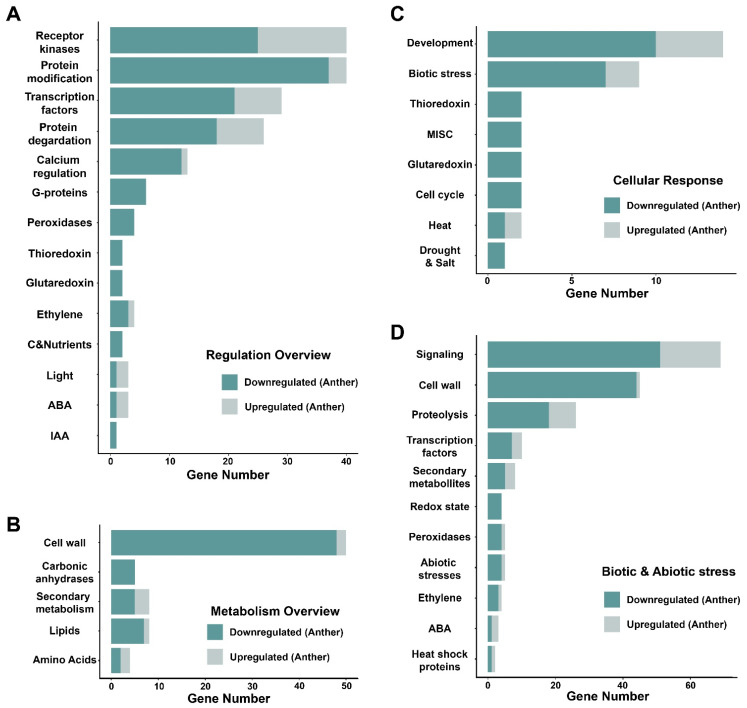
MapMan analysis of DEGs in anther. Each graph is the degree of enrichment for the MapMan pathway. (**A**) is the regulation overview, (**B**) is the metabolism overview, (**C**) is the cellular response, and (**D**) is the biotic and abiotic stress among the MapMan pathway. The x-axis represents the gene number, and the y-axis represents the molecular function registered in MapMan. Dark green indicates the anther downregulated genes, and light green is the anther upregulated genes.

MapMan analysis also supported the results of GO analysis (Figure 3 and Figure S2). Using the MapMan toolkit, we analyzed four overviews associated with mature anther DEGs: regulation, metabolism, cellular response overview, and biotic and abiotic stress overview. Of the 274 anther upregulated genes, 40 were mapped to the regulation overview. Furthermore, 18 signaling receptors, 8 TF, 8 genes related to protein degradation, 3 genes related to post-translational modification, and 3 genes related to hormone metabolism were identified (Figure 3A). The signaling receptors included domain unknown functions and glycoprotein; TF included AP2/EREBP, bHLH, MYB, and WRKY; protein degradation-related genes included a serine protease, ubiquitin, and E3 ligase. Among 658 anther downregulated genes, 137 were mapped to the regulation overview, and there were 25 signaling receptors, 23 TF, 12 calcium signaling-related genes, and 6 G-protein signaling-related genes. Signaling receptors included leucine-rich repeat and Catharanthus roseus-like RLK1 (CrRLK1L), TF included AP2/EREBP, C2H2 zinc finger, MADS, MYB, and NAC, and calcium signing-related genes included calcium-dependent protein kinase (CPK) and calmodulin-like (CML). Additionally, anther upregulated genes did not have a significantly enriched feature in the metabolism overview (Figure 3B). However, 45 genes of the anther downregulated genes were closely related to cell wall modification and organization, including 11 PMEs and 8 pectate lyases. In addition, anther downregulated genes included four peroxidase precursors, two glutaredoxins, and two thioredoxins (Figure 3C,D), indicating that the ROS signaling process might play an important role in the downstream pathways of *OsMADS62*, *OsMADS63*, and *OsMADS68*.

### 2.5. Literature Searches of the Downregulated Genes in OsMADStri Suggest Their Potential Function for Future Studies

Among the 658 anther downregulated genes, 324 were in funricegenes, of which 52 are registered with MapMan (Table 1). Cell wall-related genes were the most enriched, with 11 *OsPMEs* (*OsPME10*, *OsPME12*, *OsPME14*, *OsPME16*, *OsPME23*, *OsPME26*, *OsPME27*, *OsPME32*, *OsPME33*, *OsPME35*, *OsPME41*), and cellulose synthase-like D 3 (*CSLD3*). Regarding calcium regulation, five *OsCPKs* (*OsCPK14*, *OsCPK21*, *OsCPK25*, *OsCPK26*, *OsCPK27*), four *CMLs* (*CML2*, *CML3*, *CML28*, *CML32*), and Calcium-dependent protein kinase 2 (*CDPK2*) were included. Additionally, *SWEET5*, which is involved in sugar and ion transport, and genes encoding G-protein coupled complex, such as *OsRopGEF2, OsRopGEF8,* and *OsRopGEF3,* also belonged to the anther downregulated genes. The function of *OsRopGEF* in late pollen development has been previously reported [31]. The double loss-of-function mutant of *OsRopGEF2* and *OsRopGEF8* showed a phenotype with reduced fertility [31].

### 2.6. Genes Expected to Be Directly Regulated by OsMADS among Anther Downregulated Genes

**Figure 4:**
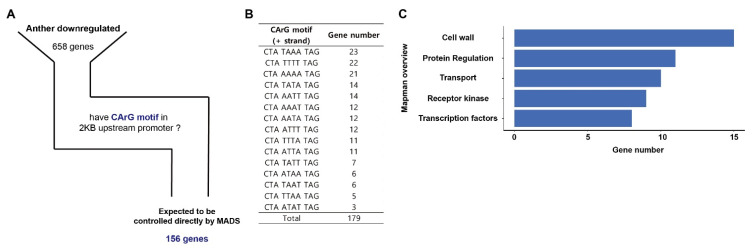
Screening and analyzing the CArG Cis-regulatory elements motif in the promoter of anther downregulated genes. (**A**) Of the 658 anther downregulated genes, 156 genes had CArG motifs in 2kb (2000 base-pair) upstream of the promoter regions. The upstream were numbered based on the adenine of the start codon. (**B**) Types and frequencies of CArG motifs existed in the promoter of anther downregulated genes. (**C**) MapMan enrichment analysis for 156 DEGs that had CArG motif in the promoter region. The x-axis means gene number, and the y-axis represents the type of overview registered in MapMan.

We identified genes that are directly regulated by three *OsMADS* among the anther downregulated genes. Since the MADS TF specifically binds to the CArG motif, genes having at least one CArG motif in the promoter of anther downregulated genes were selected. One hundred and fifty-six genes had a CArG motif in the 2 kb upstream promoters since their expression was decreased by *OsMADStri* (Figure 4A). The CArG motif consisted of ten base pairs, and DEGs having CTATAAATAG in the promoter were the most; however, DEGs with CTAATATTAG were the least (Figure 4B). When MapMan overview analysis was performed on the 156 genes, 15 were related to cell wall modification and organization, 11 to protein regulation, 9 to receptor kinases, 8 to TF (Figure 4C and Table 2). Finally, this result suggests that *OsMADS62*, *OsMADS63*, and *OsMADS68* can directly regulate candidate genes related to protein regulation, transcriptional regulation, kinase mediating signaling, and cell wall modification and organization pathways.

### 2.7. Starch Metabolism-Related Genes Whose Expression Is Significantly Reduced in OsMADStri

**Figure 5:**
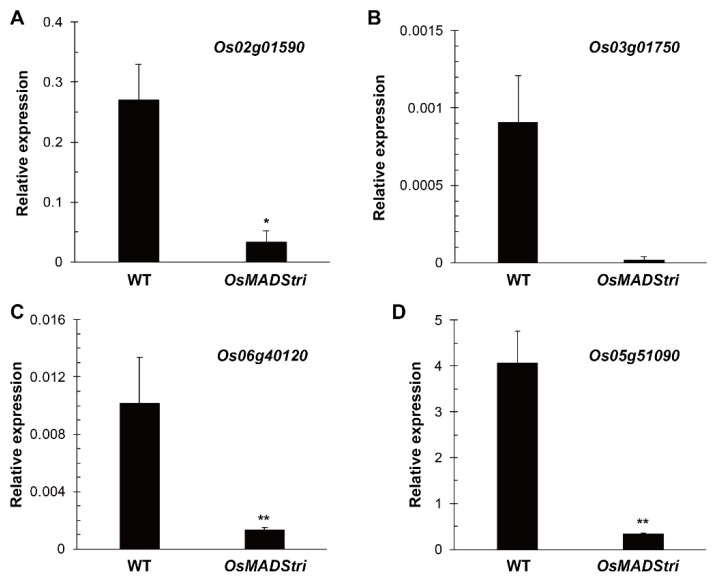
Verifying the expression patterns of four genes (**A**) LOC_Os02g01590, (**B**) LOC_Os03g01750, (**C**) LOC_Os06g40120 and (**D**) LOC_Os05g51090 related to starch accumulation among downregulated genes. The expression value of those genes was verified by RT-qPCR. *OsUBI5 (LOC_Os01g22490)* was used as an internal control. Error bars indicate the standard deviation of three biological replicates. *p*-value calculated by one-way Analysis of Variance (ANOVA) with repeated measurements using Tukey’s pairwise comparison test. *, p < 0.1; **, p < 0.01.

A mature pollen grain of *OsMADStri* was not stained with $I_{2}KI$ dye. Since $I_{2}KI$ stains starch among polysaccharides in plants, we assume that the mature pollen grain of *OsMADStri* might have defects in the starch accumulation. Therefore, we tried to find genes related to starch function among downregulated genes in *OsMADStri*. First, using the funricegene database, which provides information on rice genes with known functions [32], 132 genes were selected, including starch synthase, 14-3-3 protein, hexokinase, pyruvate kinase, and sucrose transporter. Four genes (*LOC_Os02g01590*, *LOC_Os03g01750*, *LOC_Os05g51090*, *LOC_Os06g40120*) were selected for further analyses because they have a significant expression level in WT mature anther but are extremely downregulated in OsMADStri. The largest was the log_2_FC (*OsMADStri* anther/WT anther, fold change) for *LOC_Os05g51090*, a *SWEET* gene well known as a sugar transporter. The log_2_FC value was −3.0. The log_2_FC in the other three genes, *LOC_Os02g01590* encoding *OsVIN2*, *LOC_Os03g01750* encoding polysaccharide binding phosphatase, and *LOC_Os06g40120* encoding SPX-domain-containing protein, were about −2.0. A similar tendency of reduction was confirmed in expression verification using real-time quantitative reverse transcription-polymerase chain reaction (RT-qPCR) (Figure 5). The expression value of *LOC_Os02g01590* was 0.27 at WT but decreased nine times to 0.03 at *OsMADStri*. Moreover, the expression value of LOC*_Os03g01750* decreased 49.4 times, and the expression value of *LOC_Os06g40120* decreased 7.87 times, and the expression value of *LOC_Os05g51090* was 4.06 at WT but decreased about 12 times to 0.33 at *OsMADStri*.

## 3. Discussion

Based on a previous study, we showed that functional redundancy among *OsMADS62*, *OsMADS63*, and *OsMADS68* might exist because *OsMADS68RNAi*, *OsMADS62RNAiosmads63,* and *OsMADS68RNAiosmads63* did not show a complete pollen defect [30]. Additionally, all observations and assays in the study were conducted in heterozygotes, so the molecular and genetic investigation was highly restricted [30]. In addition, studies on genes that function in the pollen maturation process have many limitations. The mutant that has a defect in pollen maturation and tube germination processes cannot participate in fertilization, so it is impossible to obtain homozygous mutants except by using gene-editing techniques [33,34]. Using the CRISPR-Cas9 system, we simultaneously knocked out three *OsMADS* (*OsMADS62*, *OsMADS63*, *OsMADS68*) highly expressed in mature pollen grains. Then, we generated the homozygous mutants for all three genes, *OsMADStri*; furthermore, *OsMADStri* shows a complete male-sterilizing phenotype. This result indicates that multiple gene-editing systems can easily overcome functional redundancy among three pollen-preferred *OsMADS* genes. Next, we analyzed the transcriptome data using the mature anther samples of *OsMADStri*, compared to the corresponding wild-type anther. It is ideal to collect only pollen grains from mutant and wild type for transcriptome analysis, but our transcriptome analysis had technical limitations because the number of *OsMADStri* mutant is not sufficient to collect the suitable number of samples for the transcriptome analysis. Therefore, we tried to enrich the target tissue by collecting the mutant anthers. Thereafter, the technical problem was supplemented through transcriptomic data of wild-type pollen grains and mature anthers which we previously produced [35]. By applying this transcriptome data to the *OsMADStri* DEGs isolated in this study, we confirmed that most of these DEGs were also preferentially expressed in pollen grains (Figure 2A) Furthermore, by analyzing the global transcriptome of the mature anther of *OsMADStri*, we identified many potential downstream genes or biological processes regulated by *OsMADS*. Among them, cell wall modification and clathrin coat assembly were the most enriched in anther downregulated genes. This suggests that *OsMADS62*, *OsMADS63*, and *OsMADS68* might regulate pollen tube growth through cell wall modification and intercellular trafficking of molecules or enzymes during tube growth.

### 3.1. OsMADStri Is Defective in the Late Maturation Stage of Pollen

Under $I_{2}KI$ staining, pollen grains in the *OsMADStri* were partially stained, and starch did not accumulate sufficiently in the pollen grains. Furthermore, *OsMADStri* showed abnormal intine and pectin staining, and it could not germinate. This was significantly different with ruptured phenotypes from other male-sterile mutant lines [16,33,34]. Most male-sterile mutants have a defect in tapetum by unusual programmed-cell death (PCD), which affects early pollen development consisting of young microspore, vacuolated pollen, and binucleate pollen, particularly pollen outer wall development, and causes problems in the entire pollen grains of the mutants [36,37,38]. Likewise, mutant lines that have problems before the first mitosis stage show a shrunken and burst pollen grain phenotype due to abnormal pollen wall formation [36,37,38]. Starch filling occurs in the late bicellular microspore stage after the first mitosis [8]. Moreover, the expression of these *OsMADS* was very low in the early pollen development consisting of a tetrad (stage 8), young microspores (stage 9), and vacuolated microspores (stage 10) stages, and the expression patterns were confirmed by qRT-PCR. Based on these expression patterns, we expect that three *OsMADS* genes play important roles in the pollen maturation process or the later processes. Because *OsMADStri* pollen cannot induce a germination and tube elongation step, it seems that *OsMADStri* already had a defect at the pollen maturation stages. Rather, the phenotype of *OsMADStri* was similar to that of the *Rice Immature Pollen 1 (RIP1)* mutant (*rip1*). *RIP1* functions in the pollen maturation stage, and its mutations had male-sterile phenotype and abnormalities in the starch accumulation and intine layers [39]. There was no difference in WT and *rip1* in the pollen grain size, the internal vacuole did not disappear, and the cytoplasmic density remained low in *rip1* [39]. However, since there was no significant difference in the expression of *RIP1* in *OsMADStri*, RIP1 functions upstream of the three *MADSs,* or in the different pathway with that of the three *MADSs*, further study is required.

### 3.2. The Low Expression of OsVIN2 and SPX Genes in OsMADStri Would Prevent Starch Accumulation

Expression of three *OsMADS* with specifically high gene expression in pollen grains were observed in pollen but not in anther walls [30]. It had very low expression at stage 10, and as they reach stage 11–12, the expression gradually increased [30]. Therefore, it can be assumed that these three MADS TFs are associated with the development of the late stages of pollen maturation. Furthermore, *OsMADStri* was defective in starch accumulation and was well correlated with the developmental expression feature. In the case of control plants, starch highly accumulated in pollen grains at the mature (tricellular) stage [8]. Furthermore, our study confirmed the result in the homozygous mature pollen of *OsMADStri*. To explain the molecular base, we listed downregulated genes in *OsMADStri* mature pollen grains and discovered that four genes were related to the starch metabolism pathway. Unexpectedly, they did not have specifically high expression in pollen grains. The four genes might be mainly involved in sugar transport and flux with SWEET, *OsVIN2*, polysaccharide binding phosphatase, and SPX-domain connecting protein. *OsVIN2* is a well-known gene that regulates the size and weight of seeds due to the flux regulation of sucrose [9,40]. In addition, *OsSPX1* regulates the expression of genes encoding hexokinase and sugar transporter [41]. *LOC_Os06g40120*-encoding SPX-domain that connects protein should have similar functions in mature pollen with *OsSPX1*. Starch is stored in the late stage of pollen maturity, and for this process, sugar should be provided through the anther wall and tapetum [42,43]. However, our RNA-seq analysis revealed that *OsMADStri* had the reduced expression of genes related to sugar transport and sucrose flux. Therefore, it might cause insufficient starch accumulation during the pollen maturation, resulting in the male sterility of *OsMADStri*.

### 3.3. PME Is Related to Cell Wall Organization and It Controls the Concentration of Calcium Ions Inside Pollen Grains

*OsMADStri* pollen grains exhibited abnormal staining in Ruthenium red and Calcofluor white. Ruthenium red stains acid polysaccharides, a constituent of pectin, making it possible to check whether the pectin layer of pollen grains is normal [44]. Calcofluor white stains cellulose and is used to identify the intine layer of pollen grains [33,45]. In addition, since the intine layer of the pollen wall is composed of cellulose and pectin, we can say that of *OsMADStri* is abnormal [46]. The size of *OsMADStri* pollen grain was similar to that of WT pollen; it was the form of an intact sphere, and the anther dehiscence occurred normally. Additionally, we tried to determine the molecular reason for this abnormality in the *OsMADStri* mutant through RNA-seq analysis. The genes related to the cell wall synthase pathway, vesicle secretion, and calcium signaling were significantly reduced in *OsMADStri*. *PME* was the most obvious gene family associated with anther downregulated genes in *OsMADStri*. 11 *OsPMEs* (*OsPME10, OsPME12*, *OsPME14*, *OsPME16*, *OsPME23*, *OsPME26*, *OsPME27*, *OsPME32*, *OsPME33*, *OsPME35*, *OsPME41*) were downregulated in *OsMADStri*. PME demethylesterifies pectin, hardens cell walls, and mediates the interactions between calcium ions and pectin [16,47]. Demethylesterified HG by PME allows ionic bonds to be formed between calcium ions and carbonyl groups of HG, through which external calcium ions can pass the intine to activate calcium signaling [14,47,48]. After hydration, calcium ions can be accumulated in the pollen grain, especially where the tube emerges, and it remains at a high concentration until the germination begins [49]. Calcium ions cannot pass through the pollen wall because of the low expression of the 11 *OsPME* genes in *OsMADStri*, and it might cause an abnormality in calcium signaling. However, even if the calcium ion penetrates the pollen grain, it might be difficult for calcium signal transduction to occur. This is because the expression of kinases, such as *OsCPK, CML, CDPK*, also decreased in *OsMADStri*. Additionally, *OsCPK21* is known to be involved in the late-stage development of pollen [41]. The expression of the three *OsMADS* analyzed in this study was decreased in the loss-of-function mutant of *OsCPK21*, and *OsCPK21* in *OsMADStri* was also decreased by nine times. This indicates that *OsCPK21* and three *OsMADS* genes may indirectly mutually affect the transcriptional regulation [41].

### 3.4. A Model for Downstream Genes Transcriptionally Affected by the OsMADS62, OsMADS63, and OsMADS68 Genes

**Figure 6:**
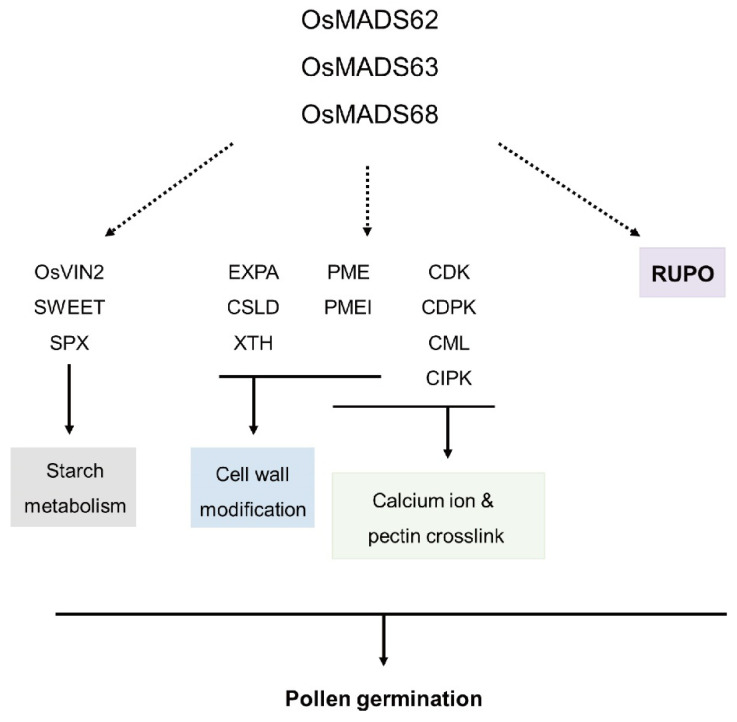
The model of downstream genes in three *OsMADS*.

First, when downregulated genes in *OsMADStri* were analyzed, they could be grouped into three main groups. The first was DEGs involved in starch metabolism, including *OsVIN2*, *SWEET*, and *SPX-domain containing protein* gene. Second, DEGs involved in cell wall synthesis include *EXPA*, *CSLD*, *XTH*, and 11 *OsPMEs*. Finally, *PME, CDK, CDPK, CML,* and *CIPK* are involved in calcium ion-meditating signaling pathways and pectin crosslink in the pollen grain. More interestingly, the *Ruptured Pollen tube* (*RUPO*), belonging to CrRLK1L, a plant-specific acceptor-like kinase, was identified as a downregulated gene with −6.78 log_2_FC in *OsMADStri*. Additionally, RUPO is located in the cell membrane and vessel of the apical region of the pollen tube and plays a role as a regulator of potassium carriers [50]. Altogether, a transcriptional regulation model mediated by *OsMADS62*, *OsMADS63*, *OsMADS68* is suggested in Figure 6. However, our transcriptome work has a limitation: exactly how DEGs are affected by each of *OsMADS62*, *OsMADS63*, and *OsMADS68* or combination among them is unclear. Furthermore, since OsMADS TF forms a heterodimer for the function, it might be required to analyze the interaction between the promoter of key DEGs and the combination of three OsMADS proteins through further studies. Unlike dicotyledonous plants, such as *Arabidopsis thaliana*, rice has one ovule in each flower. Since it is impossible for mutant pollen grain to compete with the wild-type pollen grain and complete the fertilization process, there was a limitation in that heterozygous lines available through current biotechnology or breeding techniques had to be used. As a result, few studies have been published. With the CRISPR/Cas9 system, it is possible to produce homozygous mutants which enable to carrying out detailed genetic and molecular analyses on pollen maturation or pollen tube germination in rice. The study of transcriptional analysis using the *OsMADStri* in this study is expected to be a very useful resource for understanding the overall regulatory process for the maturation and germination of rice pollen mediated by *OsMADS62*, *OsMADS63*, and *OsMADS68*.

## 4. Materials and Methods

### 4.1. Plant Materials and Growth Conditions

WT rice (*O. sativa japonica* cv. Dongjin) seeds were sterilized with 50% of sodium hypochlorite and washed with distilled water and germinated on Murashige and Skoog media (pH 5.7) under a growth chamber in 7 days (28/25 °C day/night) [51]. The seedlings were grown in the greenhouse for three weeks and transferred to a paddy field at the Kyung Hee University. In the case of *OsMADStri*, since the mutant showed a completely sterile phenotype, the transgenic T0 plant was ratooned and maintained for two years in the greenhouse and paddy field. Transplanting was conducted at the end of May 2020 and 2021, and seeds were harvested at the end of October.

### 4.2. Nucleic Acids Extraction and RT-qPCR

For genotyping analysis, flag leaves of transgenic plants were sampled, frozen in liquid nitrogen, and ground using a Tissue-Lyser II (Qiagen, Hilden, Germany). DNA was extracted using the CTAB (cetyltrimethylammonium bromide)-chloroform method [52]. In addition, sequencing analysis for genotyping was performed by Macrogen (https://dna.macrogen.com, accessed on 4 January 2021), using the BigDye Terminator v3.1 cycle sequencing kit (Applied Biosystems). For total RNA extraction, WT and mutant anther tissues were sampled by freezing in liquid nitrogen. After grinding the frozen sample with liquid nitrogen using a pestle and mortar (CoorsTek 60310), total RNA was extracted using TRIzol buffer and 1-bromo-3-chloropropane (BCP) and purified using the RNeasy plant mini kit (Qiagen, Hilden, Germany). Finally, the complementary DNA (cDNA) was synthesized using SuPrime Script RT premix (GeNet Bio). To identify the tissue-specific expression by quantitative real-time PCR (RT-qPCR), we used the Roter-Gene Q instrument system (Qiagen, Hilden, Germany) and the internal control for rice ubiquitin 5 (*OsUbi5, LOC_Os01g22490*), as previously reported [53]. RT-qPCR was performed with three independent biological replicates. A previously reported method was used to calculate the relative transcript levels and fold change [54]. All RT-qPCR primers used in our experiments are listed in Table S1.

### 4.3. Vector Construction and Plant Transformation

First, to design the guide RNA for CRISPR-Cas9 vector cloning, we selected two target regions using the CRISPRdirect software, and the synthesized oligo dimer was ligated to the pRGEB32 binary vector [55]. Next, the modifying plasmid vector was amplified in *Escherichia coli*; TOP10, and then transformed into the *Agrobacterium tumefaciens*, *LBA4404*. Induced rice callus was co-cultivated with *Agrobacterium* cells and incubated on a 2N6-CH30 and 2N6-BA medium in 5 to 6 weeks under 25 °C in dark conditions [56] Finally, the transgenic rice plants were regenerated in 2 to 10 weeks under 26 °C light conditions in solidified MS medium [51,56,57]. All primers used in this study are listed in Table S1.

### 4.4. Phenotypic Analysis

To calculate the in vitro germination ratio, the viable pollen grains were germinated in solid and liquid pollen germination media. The liquid pollen germination medium consists of 20% sucrose, 10% polyethylene glycol 4000 (PEG4000), 3 mM $\mathrm{Ca}{\left({\mathrm{NO}}_{3}\right)}_{2}$, 40 mg/L $H_{3}{\mathrm{BO}}_{3}$, and 10 mg/L thiamine (Vitamin B1) [33]. To make a solid germination medium, we added 1% agarose to the liquid medium. When the rice flowers reached anthesis, the fully mature pollen grains were collected in germination media immediately. Afterward, pollens on the germination media were incubated at 28 °C for about 30 min. We kept the humidity to prevent the germination medium from drying and observed the pollens using a BX61 microscope (Olympus, Tokyo, Japan). The pollen germination state and tube length were measured using Image J software [58]. More than 100 pollens were analyzed daily for a week. Ruthenium red and Calcofluor white were used for pectin, intine staining. All histochemical staining was incubated at room temperature for 15 min [31,33]. The stained pollen grains were observed using the Olympus BX61 microscope.

### 4.5. RNA Sequencing and Bioinformatic Analysis

We sampled the anthers that contained pollen at tricellular stages. Pollens at different developmental stages were distinguished according to the size of the anther and the length of the filaments, referring to previous studies [59]. RNA-seq was performed on the Illumina platform in Macrogen (Korea). Raw data were rearranged using Cutadapt, mapped to the MSU7 reference genome, and then normalized by raw read counting to FeatureCounts and DESeq2 using the R package, following this reference [33,60,61]. GO enrichment features were selected by fold change threshold of >2, and the *p*-value < 0.05. The GO plot is visualized using the R studio ggplot2 package. Upregulated genes and downregulated genes in the anther were inputted into the MapMan program. Regulation overview, metabolism overview, cellular response, and biotic and abiotic stress were selected for overview mapping. The bar graph was visualized using the R studio ggplot2 package. CAFRI-rice was used for expression analysis [62]. K-means clustering analysis was conducted by MultiExperiment Viewer software [63,64]

## Figures and Tables

**Table 1:** The GO annotation and MapMan analysis of known genes in anther downregulated genes of OsMADStri.

DEG	Locus	Gene Name	MapMan	GO Number	GO Name
Down	LOC_Os12g43700	PR1-121	Biotic stress	Non	Non
Down	LOC_Os08g23460	OsSTA209		Non	Non
Down	LOC_Os12g30150	OsCPK27	Calcium regulation	GO:0007186	G-protein coupled receptor protein signaling pathway
Down	LOC_Os10g27170	OsSTA242		Non	Non
Down	LOC_Os12g03970	OsCPK26		GO:0006468	protein amino acid phosphorylation
Down	LOC_Os11g04170	OsCPK25		GO:0006468	protein amino acid phosphorylation
Down	LOC_Os12g12730	CML28		GO:0001539	ciliary or flagellar motility
Down	LOC_Os08g04890	CML32		GO:0001539	ciliary or flagellar motility
Down	LOC_Os08g42750	OsCPK21		GO:0006468	protein amino acid phosphorylation
Down	LOC_Os05g41270	OsCPK14		GO:0006468	protein amino acid phosphorylation
Down	LOC_Os01g59360	CDPK2		GO:0006468	protein amino acid phosphorylation
Down	LOC_Os11g03980	CML2		GO:0001539	ciliary or flagellar motility
Down	LOC_Os12g03816	CML3		GO:0001539	ciliary or flagellar motility
Down	LOC_Os11g45720	OsPME33	Cell wall	GO:0042545	cell wall modification
Down	LOC_Os12g37660	OsPME35		GO:0042545	cell wall modification
Down	LOC_Os12g36040	EXPA26		GO:0007047	cellular cell wall organization
Down	LOC_Os04g54850	OsPME16		GO:0042545	cell wall modification
Down	LOC_Os11g43830	OsPME32		GO:0042545	cell wall modification
Down	LOC_Os04g39590	FLA14		Non	Non
Down	LOC_Os07g49100	OsPME23		GO:0042545	cell wall modification
Down	LOC_Os08g25710	CSLD3		GO:0030244	cellulose biosynthetic process
Down	LOC_Os09g26360	OsPME27		GO:0042545	cell wall modification
Down	LOC_Os04g38560	OsPME14		GO:0042545	cell wall modification
Down	LOC_Os03g28090	OsPME12		GO:0042545	cell wall modification
Down	LOC_Os03g18860	OsPME10		GO:0042545	cell wall modification
Down	LOC_Os08g34910	OsPME26		GO:0042545	cell wall modification
Down	LOC_Os02g03550	XTH26		GO:0005975	carbohydrate metabolic process
Down	LOC_Os09g37360	OsPME41		GO:0042545	cell wall modification
Down	LOC_Os08g13980	OsSTA207		GO:0005975	carbohydrate metabolic process
Down	LOC_Os02g26320	FLA20		Non	Non
Down	LOC_Os09g22090	OsSTA225		GO:0005975	carbohydrate metabolic process
Down	LOC_Os06g08810	OsSTA172		GO:0045226	extracellular polysaccharide biosynthetic process
Down	LOC_Os11g08400	OsSTA253	Development	Non	Non
Down	LOC_Os10g35930	OsPLIM2c		Non	Non
Down	LOC_Os03g27610	OspPLAIIbeta		GO:0006629	lipid metabolic process
Down	LOC_Os12g07874	OsSTA276		GO:0006412	translation
Down	LOC_Os05g51090	SWEET5		GO:0006813	potassium ion transport
Down	LOC_Os01g55520	OsRopGEF8	G-proteins	Non	Non
Down	LOC_Os05g48640	OsRopGEF2		Non	Non
Down	LOC_Os02g17240	OsRopGEF3		Non	Non
Down	LOC_Os06g45240	OsSTA177	Receptor kinases	GO:0006468	protein amino acid phosphorylation
Down	LOC_Os05g38980	OsNOX4	Redox state	GO:0055114	oxidation reduction
Down	LOC_Os01g40400	HDA701	Transcription factors	GO:0016575	histone deacetylation
Down	LOC_Os11g32960	OsSTA261		GO:0007275	multicellular organismal development
Down	LOC_Os10g32900	OsCCT34		Non	Non
Down	LOC_Os04g38770	MADS61		GO:0045449	regulation of transcription
Down	LOC_Os04g46670	OsSTA129		Non	Non
Down	LOC_Os01g11350	OsbZIP02		GO:0006355	regulation of transcription, DNA-dependent
Down	LOC_Os11g43740	MADS68		GO:0045449	regulation of transcription
Down	LOC_Os08g38590	MADS62		GO:0045449	regulation of transcription
Down	LOC_Os12g22630	OsSTA279		GO:0045449	regulation of transcription
Down	LOC_Os07g31830	OsSTA196		Non	Non

**Table 2:** The promoter analysis of anther downregulated genes in OsMADStri mutant.

Locus ID	Gene Name	GO	Functions	log2 FC	Sequence
LOC_Os11g45720	OsPME33	cell wall modification	Cell wall	−19.25	CTAAAATTAG
LOC_Os01g55440	CIPK30	signal transduction	Protein modification	−17.43	CTAATTTTAG
LOC_Os05g29740	OsPMEI23	homoiothermy	PME	−16.10	CTAAAATTAG
LOC_Os05g11790	CIPK20	signal transduction	Protein modification	−15.79	CTAAAAATAG
LOC_Os08g07600	OsPROPEP2	Non	Signaling	−13.86	CTATAATTAG
LOC_Os11g08400	OsSTA253	Non	Development	−13.50	CTATAAATAG
LOC_Os10g35930	OsPLIM2c	Non	Development	−13.16	CTATATTTAG
LOC_Os01g20970	OsPMEI3	Non	PME	−11.68	CTAAAATTAG
LOC_Os11g32960	OsSTA261	multicellular organismal development	Transcription factor	−11.54	CTAAAAATAG
LOC_Os02g02450	YSL7	transport	Transport	−11.50	CTAAAAATAG
LOC_Os02g58660	OsSTA85	cation transport	Transport	−10.99	CTATATATAG
LOC_Os10g32900	OsCCT34	Non	Transcription factor	−10.00	CTATAATTAG
LOC_Os08g23130	OsOPT5	Non	Transport	−9.76	CTAAAAATAG
LOC_Os12g12860	OsCPK29	glycogen biosynthetic process	Protein modification	−9.51	CTAAATTTAG
LOC_Os01g13710	YSL1	pathogenesis	Transport	−9.31	CTAAAAATAG
LOC_Os02g33840	OsSTA64	Non	Protein modification	−9.17	CTAAAAATAG
LOC_Os02g09450	OsGDPD11	lipid metabolic process	Lipid	−9.07	CTAAAATTAG
LOC_Os05g38980	OsRBOH4	oxidation reduction	Redox	−8.87	CTAAATATAG
LOC_Os02g33740	OsSTA63	Non	Protein modification	−8.47	CTAATTATAG
LOC_Os03g10550	OsSTA94	Non	Non	−8.24	CTATTTATAG
LOC_Os06g06430	OsSTA169	Non	Non	−7.46	CTAAATATAG
LOC_Os02g31950	OsSTA59	Non	Non	−5.46	CTAAAAATAG
LOC_Os02g51730	OsSTA78	Non	Redox	−5.46	CTAATAATAG
LOC_Os02g02460	YSL8	transport	Transport	−4.85	CTAAATTTAG
LOC_Os03g16840	OsSTA95	Non	Non	−3.68	CTAATATTAG
LOC_Os05g20150	OsMTD4	protein amino acid phosphorylation	Signaling	−3.51	CTAATTTTAG
LOC_Os02g03550	XTH26	carbohydrate metabolic process	Cell wall	−3.30	CTAATTTTAG
LOC_Os02g01990	OsCCT02	Non	Non	−3.06	CTATTTATAG
LOC_Os05g28530	OsGRL10	cell redox homeostasis	Redox	−3.00	CTATATTTAG
LOC_Os08g13980	OsSTA207	carbohydrate metabolic process	Cell wall	−2.86	CTAAAAATAG
LOC_Os12g07700	ISC14	iron-sulfur cluster assembly	Protein modification	−2.78	CTAAATATAG
LOC_Os06g08810	OsSTA172	carbohydrate metabolic process	Cell wall	−2.16	CTATATTTAG
LOC_Os07g31830	OsSTA196	Non	Transcription factor	−2.13	CTATTAATAG
LOC_Os01g03640	OsLPR5	oxidation reduction	Redox	−2.02	CTATTTATAG

## Data Availability

All data are within the manuscript and its Supplementary files. Raw sequencing data and metadata have been deposited in ArrayExpress (https://www.ebi.ac.uk/arrayexpress/experiments/E-MTAB-11246, accessed on 2 December 2021), with accession number E-MTAB-11246.

## Supplementary Materials

The following are available online at https://www.mdpi.com/article/10.3390/ijms23010239/s1.
