# Supplementary material for: Transcriptome Analysis of Triple Mutant for OsMADS62, OsMADS63, and OsMADS68 Reveals the Downstream Regulatory Mechanism for Pollen Germination in Rice (Oryza sativa)
Source: Int J Mol Sci. 2021 Dec 27;23(1):239. doi: 10.3390/ijms23010239 (PMC8745200; doi:10.3390/ijms23010239)
Supplement: Supplementary file 1 [file ijms-23-00239-s001.zip › ijms-1514936-supplementary.pdf]

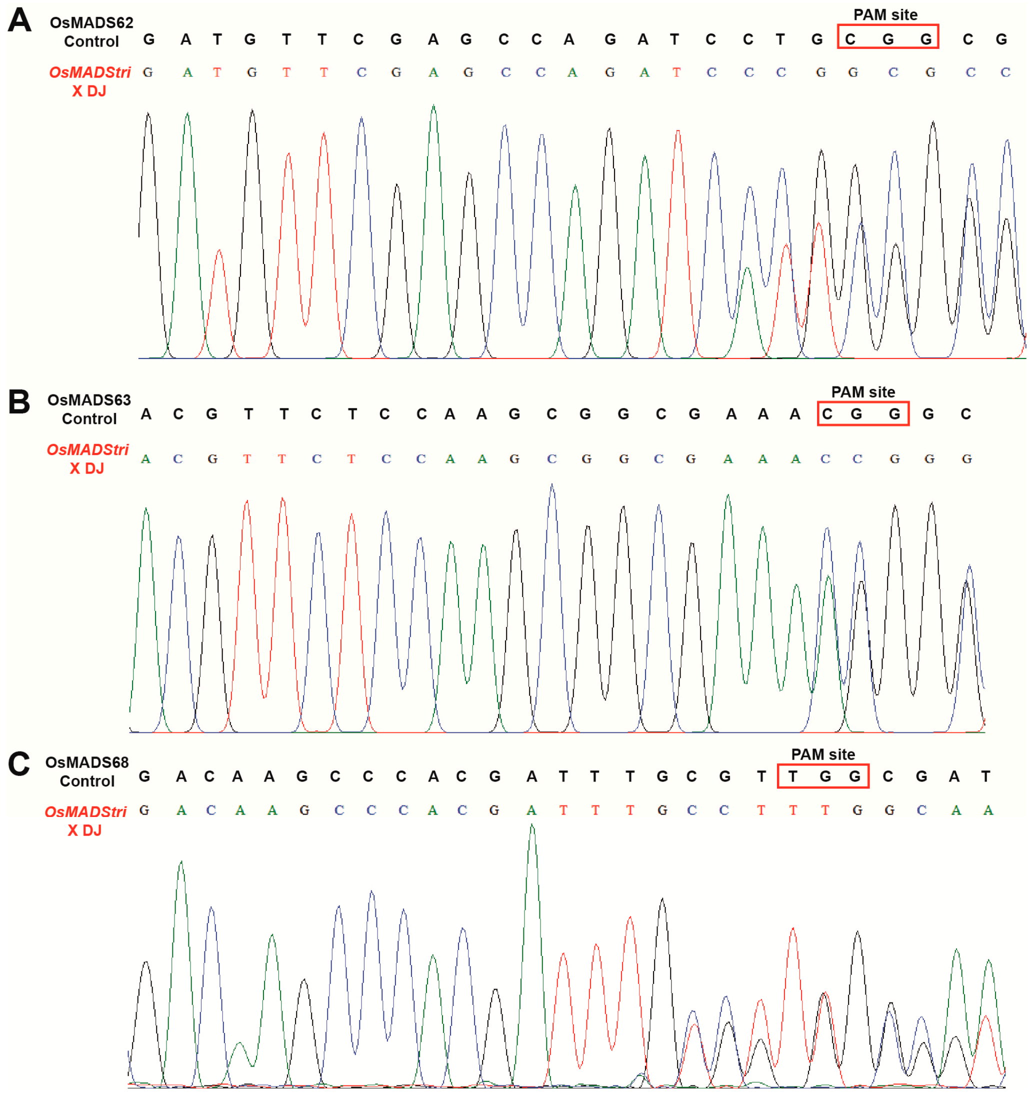

Figure S1: Sequence comparison of *OsMADS62*, *OsMADS63*, and *OsMADS68* between WT (DongJin, DJ) and *OsMADStri* mutants.

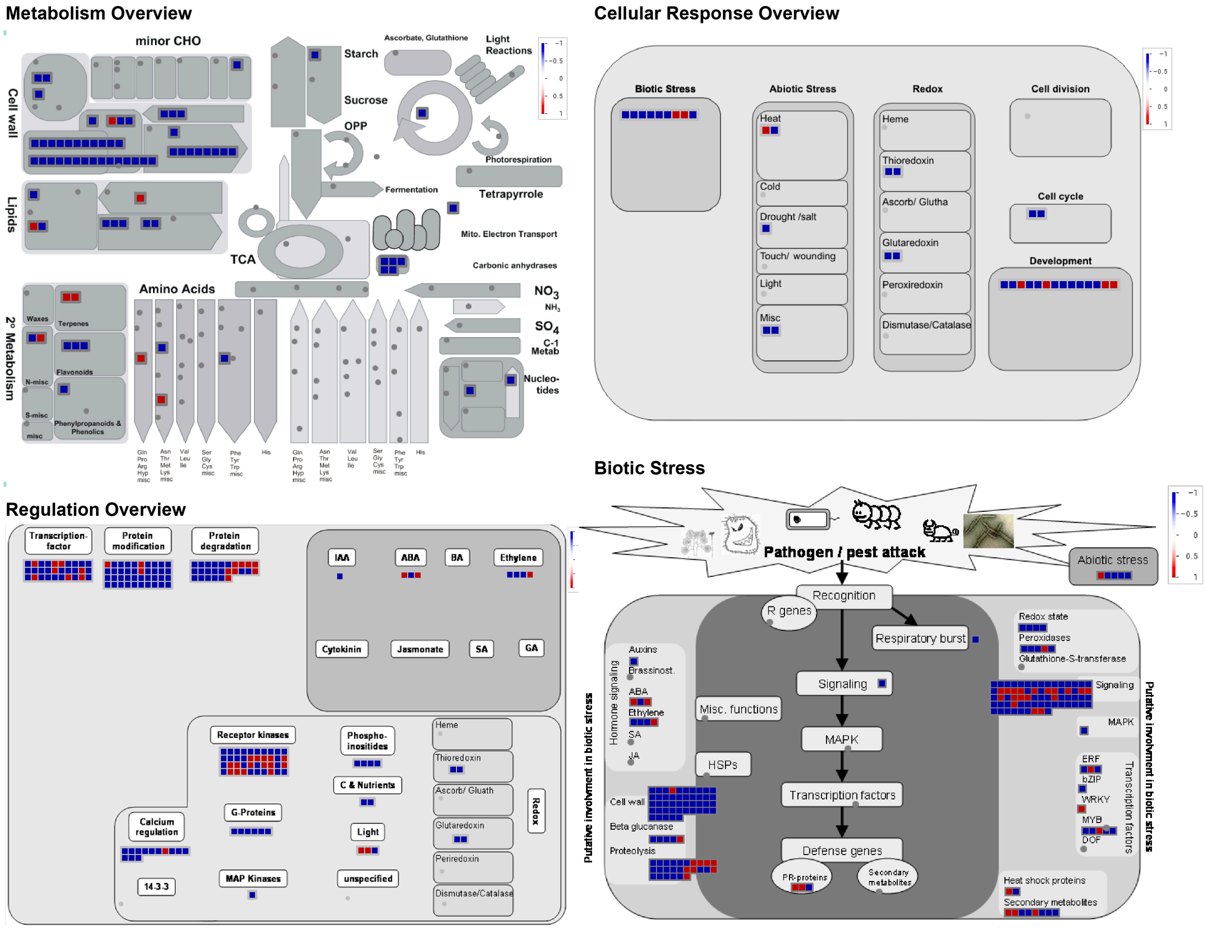

Figure S2: Detailed results of MapMan analysis

Supplementary Table S1. Primer sequences used in this study.		
**Primer names**	**Primer sequences**	**Purpose**
OsMADS62_Multiple_Cas_F	TAGGTCTCC AGCCAGATCCTG gttttagagctagaa	CRISPR-Cas9
OsMADS62_Multiple_Cas_R	CGGGTCTCA GGCTCGAACATC tgcaccagccggg	CRISPR-Cas9
OsMADS63_Multiple_Cas_F	TAGGTCTCC CAAGCGGCGAAA gttttagagctagaa	CRISPR-Cas9
OsMADS63_Multiple_Cas_R	CGGGTCTCA CTTGGAGAACGT tgcaccagccggg	CRISPR-Cas9
OsMADS68_Multiple_Cas_F	TAGGTCTCC CACGATTTGCGT gttttagagctagaa	CRISPR-Cas9
OsMADS68_Multiple_Cas_R	CGGGTCTCA CGTGGGCTTGTC tgcaccagccggg	CRISPR-Cas9
OsUBI5_qRT_F	GCACAAGCACAAGAAGGTGA	RT-qPCR
OsUBI5_qRT_R	GAATCGACTGGAGGGAAGCC	RT-qPCR
OsMADS62_qRT_F	CATCCAGAATCGGGAGG	RT-qPCR
OsMADS62_qRT_R	GTCGACCTCGCTGGATGTG	RT-qPCR
OsMADS63_qRT_F	CGGTGTACGACACGATGAGA	RT-qPCR
OsMADS63_qRT_R	AGTCGTCACTCTGGTTCTGC	RT-qPCR
OsMADS68_qRT_F	CACGTACTCGAAACGGAGGT	RT-qPCR
OsMADS68_qRT_R	TATGCTGCTGTGATCGCCAA	RT-qPCR
LOC_Os02g01590_qRT_F	CCTTCTACGATCCGGCCAAG	RT-qPCR
LOC_Os02g01590_qRT_R	CTTCCGCAAAAGTACCGCCT	RT-qPCR
LOC_Os03g01750_qRT_F	GCAGCAAGATCCAGACCTTGAATA	RT-qPCR
LOC_Os03g01750_qRT_R	TTTCGGCACGACAGTGCTC	RT-qPCR
LOC_Os05g51090_qRT_F	CTACGGCCTACCCTTCATCC	RT-qPCR
LOC_Os05g51090_qRT_R	GGCGTAGGCGAAGTAGATGG	RT-qPCR
LOC_Os06g40120_qRT_F	AGCACTAACGAGGACAAGCC	RT-qPCR
LOC_Os06g40120_qRT_R	CCGCTTCGGATCTCCTTCAG	RT-qPCR
OsMADS62_Genotyping_F	CAAATTTCAGCCCAGGTGCC	Mutant genotyping
OsMADS62_Genotyping_R	CAGCTGTCGTTGCATCGAAC	Mutant genotyping
OsMADS63_Genotyping_F	ATGGACGCGGTCCGTCCACT	Mutant genotyping
OsMADS63_Genotyping_R	CGAAGAAGTTTACGTACCCT	Mutant genotyping
OsMADS68_Genotyping_F	GGTTTCGACGAATTGGACTG	Mutant genotyping
OsMADS68_Genotyping_R	GTGGAATTGGGTTCGTTCAG	Mutant genotyping

Table S1: Primer sequences used in this study.
